# Supplementary material for: Inactivation of Genes Encoding MutL and MutS Proteins Influences Adhesion and Biofilm Formation by Neisseria gonorrhoeae
Source: Microorganisms. 2019 Dec 4;7(12):647. doi: 10.3390/microorganisms7120647 (PMC6955733; doi:10.3390/microorganisms7120647)
Supplement: Supplementary file 1 [file microorganisms-07-00647-s001.pdf]

Supplementary Table S1. Primers used in the work.

Gene	Primer names	Primer sequences (5’ → 3’)
ngo0105	ngo0105For	AAGGCTTCGTGTTGCACAT
	ngo0105Rev	TATTCGGTATTGCGGTTGAA
ngo0100	ngo0100For	CAGTGCCATCAATACCCTGA
	ngo0100Rev	TGCAGCTCGAAGAAGTTGAT
ngo0237	ngo0237For	ATCGTGCTGCACAACATTTC
	ngo0237Rev	TGTATCAGGCTGAAGAACGG
ngo0572	ngo0572For	AAGGTGCGATGAAGGGTATG
	ngo0572Rev	CGCTGGTGGACTCCTTTATC
ngo0626	ngo0626For	CATTGGCGACTTTAGGCTTTG
	ngo0626Rev	ACATCACCGCCTTCTTCTTT
ngo0834	ngo0834For	GCAATACGCAATATCACGGT
	ngo0834Rev	GCAGGTGTGTTACCAGGATG
ngo0908	ngo0908For	CGCAGTACCGAAGCAGAAA
	ngo0908Rev	CCGATTTCCTGCCCTATTGAT
ngo1392	ngo1392For	AACAACCTCGGCAGCAATTT
	ngo1392Rev	CGCGTTCCGTACAGTATGTC
ngo1393	ngo1393For	ACCTCATCCAAACCGTCTTC
	ngo1393Rev	TAGAGGTGCAGTTCGGTACG
ngo1439	ngo1439For	CCAGCCGTGTATATGGGTATG
	ngo1439Rev	GCTTGTTGCCCTCTTTACTTG
ngo1535	ngo1535For	AAATCTCGGCTTTGGTATGC
	ngo1535Rev	AAATCTCGGCTTTGGTATGC
ngo1537	ngo1537For	TAGAAGCCGTGTCCGTTATG
	ngo1537Rev	GACGACGACTTGGGTTTCTT
ngo1543	ngo1543For	CTGAACGGCAATGAACAAAT
	ngo1543Rev	CCAAAGCTGTGAAGTTGAGC
ngo1549	ngo1549For	TAGAATATCCGCCCGTAAGC
	ngo1549Rev	GAAGAAACCGGACAGACCTT
ngo1577	ngo1577For	GTGAGGCTCTGATTGCATGT
	ngo1577Rev	AGTGTTGGTGATGATTGCGT
ngo1585	ngo1585For	AATGATTGTCCGCCTAAACC

	ngo1585Rev	GGTTTCCTGTGTCGGAATCT
ngo1659	ngo1659For<br>ngo1659Rev	ACGGCTTGAAAGCGAGTATC<br>ACCAGTTGGCAATACCCATAAA
ngo1682	ngo1682For<br>ngo1682Rev	GTCTGAAACGGAGGACACAA<br>GTTGGCGATGGTCGTATCTAA
ngo1683	ngo1683For<br>ngo1683Rev	AACGGGCAACTGGATTAAAG<br>GTCATCGACAAACCGATACG
ngo1822	ngo1822For<br>ngo1822Rev	TACGGCAGGTATTGCTTCAG<br>GCATGCTCATAGAACCTTGG
ngo2158	ngo2158For<br>ngo2158 Rev	GACCGATTTGCTGGATTTCT<br>CAATAGCTGCGGTATTTCCA
ngo2159	ngo2159For<br>ngo2159 Rev	TAGTCCCGCCTTATGTACCC<br>GCACGCTTCAAGTCAAACAT
16S rRNA	16S RTF<br>16S RTR	GCGTGGGTAGCAAACAGGAT<br>CGCGTTAGCTACGCTACCAAG
FIBRONE CTIN	fibronektyna for<br>fibronektyna rev	GACTCGCTTTGACTTCACCA<br>TTCAGAAGTGGCCACAAGAG
CEACAM1	CEACAM1	10025636*<br>qHsaCED0033913**
HSPG	HSPG2	10025636*<br>qHsaCID0020933**
CD46	CD46	10025636*<br>qHsaCID0013380**
VTN	VTN	10025636*<br>qHsaCED0037065**
B2M	B2M	10025636*<br>qHsaCID0015347**
HPRT1	HPRT	4326321E***<br>Hs9999909_m1****

* catalog number (BIO-RAD)

** assay ID (BIO-RAD)

*** catalog number (Thermo Scientific)

**** assay ID (Thermo Scientific)

Supplementary Table S2. Expression of specific genes in (A) *N. gonorrhoeae mutL::km* and (B) *N. gonorrhoeae mutS::km* mutant strains compared to the wild-type strain. One-letter abbreviations for the functional categories: **Cellular processes and signaling** (**D -** Cell cycle control, cell division, chromosome partitioning; **M -** Cell wall/membrane/envelope biogenesis; **N -** Cell motility; **O -** Post-translational modification, protein turnover, and chaperones; **T -** Signal transduction mechanisms; **U -** Intracellular trafficking, secretion, and vesicular transport; **V -** Defense mechanisms); **Information storage and processing** (**B -** Chromatin structure and dynamics; **J -** Translation, ribosomal structure and biogenesis; **K -** Transcription; **L -** Replication, recombination and repair); **Metabolism** (**C -** Energy production and conversion; **E -** Amino acid transport and metabolism; **F -** Nucleotide transport and metabolism; **G -** Carbohydrate transport and metabolism; **H -** Coenzyme transport and metabolism; **I -** Lipid transport and metabolism; **P -** Inorganic ion transport and metabolism; **Q -** Secondary metabolites biosynthesis, transport, and catabolism); **Poorly characterized** (**R -** General function prediction only; **S -** Function unknown).

Table S2A

**Gene** [**name**] **Protein ID**	**Description**	**Microarray mean fold change**	**RT-qPCR mean fold change**	**COG name**	**COG accession** [**category**]
**Upregulated**					
NGO0142 YP_207312.1	sugar transporter	3.73	nd	FucP	COG0738 [G]
NGO0156 YP_207326.1	hypothetical protein	1.51	nd	-	COG3298 [L]
NGO0191 [*rpsO*] YP_207358.1	protein=30S ribosomal protein S15/ Translation initiation factor IF-2	2.02	nd	RpsO	COG0184 [J]
NGO0206 YP_207371.1	ABC transporter periplasmic binding protein, polyamine/Putrescine-binding periplasmic protein	2.17	2.17	PotD	COG0687 [E]

NGO0207 YP_207372.1	hypothetical protein	1.68	nd	WcaA	COG0463 [M]
NGO0216 YP_207381.1	ABC transporter permease	1.56	2.0	ThiP	COG1178 [P]
NGO0234 YP_207399.1	coproporphyrinogen III oxidase	1.57	nd	HemN	COG0635 [H]
NGO0377 YP_207533.1	sodium/dicarboxylate transporter	1.55	nd	CitT	COG0471 [P]
NGO0574 YP_207719.1	Cah-Carbonic anhydrase	4.96	5.6	Cah	COG3338 [P]
NGO0720 AAW89440.1	phage associated protein	1.73	nd	-	-
NGO0721 YP_207853.1	phage associated protein	1.53	nd	-	-
NGO0912 YP_208020.1	succinyl-CoA ligase [ADP-forming] subunit alpha (EC 6.2.1.5)	2.37	nd	SucD	COG0074 [C]
NGO0913 [*sucC*] YP_208021.1	succinyl-CoA synthetase subunit beta	2.26	nd	SucC	COG0045 [C]
NGO1393 YP_208453.1	MafA-like adhesin/MafA2	1.78	2.7	-	-
NGO1481 YP_208531.1	malonyl-ACP O-methyltransferase	1.77	nd	UbiE	COG2226 [H]
NGO1482 YP_208532.1	hypothetical protein	1.64	nd	-	COG2830 [S]
NGO1483 YP_208533.1	8-amino-7-oxononanoate synthase	1.68	nd	BioF	COG0156 [H]
NGO1484 YP_208534.1	hypothetical protein	1.53	nd	ErfK	COG1376 [S]
NGO1485 YP_208535.1	anion transporter	1.51	nd	CitT	COG0471 [P]

NGO1504 YP_208554.1	rRNA large subunit methyltransferase L	1.52	nd	-	COG0116 [L]
NGO1535 [*murD*] YP_208583.1	UDP-N-acetylmuramoyl-L-alanyl-D-glutamate synthetase	1.76	2.45	MurD	COG0771 [M]
NGO1537 [*mraY*] YP_208585.1	phospho-N-acetylmuramoyl-pentapeptide-transferase	1.69	2.2	Rfe	COG0472 [M]
NGO1552 YP_208600.1	sodium/proline symporter, proline permease	1.60	nd	PutP	COG0591 [ER]
NGO1585 YP_208626.1	MafB-like adhesin/MafB4	2.71	2.7	-	-
NGO1593 YP_208633.1	hypothetical protein	3.15	nd	-	-
NGO1594 YP_208634.1	hypothetical protein	2.02	nd	-	-
NGO1595 YP_208635.1	hypothetical protein	2.36	nd	-	-
NGO1659 YP_208698.1	intracellular septation protein A	1.98	2.88	-	COG2917 [D]
NGO1817 [*rplQ*] YP_208846.1	50S ribosomal protein L17	1.67	nd	RplQ	COG0203 [J]
NGO1818 YP_208847.1	DNA-directed RNA polymerase subunit alpha	1.62	nd	RpoA	COG0202 [K]
NGO1820 YP_208849.1	30S ribosomal protein S11	1.96	nd	RpsK	COG0100 [J]
NGO1822 [*secY*] YP_208852.1	preprotein translocase subunit SecY	1.62	1.8	SecY	COG0201 [U]
NGO1834 [*rpsS*]	30S ribosomal protein S19	1.62	nd	RpsS	COG0185 [J]

YP_208868.1					
NGO1835 [*rplB*] YP_208869.1	50S ribosomal protein L2	1.51	nd	RplB	COG0090 [J]
NGO1878 YP_208911.1	hypothetical protein	3.04	nd	-	-
NGO1980 YP_209000.1	malate:quinone oxidoreductase	1.78	nd	-	COG0579 [R]
NGO2017 Q5F5B2.1	hypothetical protein	1.64	nd	SlyX	-
NGO2020 YP_209040.2	phosphoenolpyruvate carboxylase (PEPCase)	2.23	nd	Ppc	COG2352 [C]
NGO2022 YP_209042.1	hypothetical protein	2.15	nd	MreC	COG1792 [M]
NGO2092 YP_209105.1	iron ABC transporter substrate-binding protein	1.57	3.2	FepB<br>CeuA	COG0614 [P]<br>COG4607 [P]
NGO2094 [*groES*] YP_209107.1	co-chaperonin GroES	2.03	2.7	GroS	COG0234 [O]
NGO2148 YP_209157.1	ATP synthase F0F1 subunit alfa	1.59	2.47	AtpA	COG0056 [C]
NGO2149 YP_209158.1	ATP synthase F0F1 subunit gamma	1.66	2.34	AtpG	COG0224 [C]
NGO2150 YP_209159.1	ATP synthase F0F1 subunit beta	1.67	1.7	AtpD	COG0055 [C]
NGO2151 [*atpC*] YP_209160.1	ATP synthase F0F1 subunit epsilon	1.55	1.8	AtpC	COG0355 [C]
NGO2173 [*rpmF*] YP_209178.1	50S ribosomal protein L32	1.72	nd	RpmF	COG0333 [J]

**Downregulated**					
NGO0029 [*pyrE*] YP_207210.1	orotate phosphoribosyltransferase	-1.51	nd	PyrE	COG0461 [F]
NGO0237 YP_207402.1	N-acetyl-anhydromuranmyl-L-alanine amidase	-1.63	0.7	AmpD	COG3023 [V]
NGO0387 YP_207543.1	GTP cyclohydrolase	-1.53	nd	-	COG1469 [S]
NGO0486 YP_207636.1	phage associated protein	-2.11	nd	-	-
NGO0498 YP_207648.1	phage associated protein	-2.39	nd	-	-
NGO0507 YP_207657.1	phage associated protein	-1.69	nd	-	COG5449 [S]
NGO0512 AAW89250.1	phage associated protein	-1.86	nd	-	-
NGO0610 YP_207750.1	formamidopyrimidine-DNA glycosylase	-1.67	nd	Nei	COG0266 [L]
NGO0648 YP_207787.1	membrane protein	-1.50	nd	-	-
NGO0794 YP_207920.1	bacterioferritin A (BfrA)	-1.51	0.45	Bfr	COG2193 [P]
NGO0795 YP_207921.1	bacterioferritin B (BfrB)	-1.57	0.71	Bfr	COG2193 [P]
NGO0853 YP_207969.1	camphor resistance protein CrcB	-1.74	nd	CrcB	COG0239 [D]
NGO0925 YP_208033.1	dihydrolipoamide dehydrogenase	-1.97	nd	Lpd	COG1249 [C]
NGO0926 YP_208034.1	peroxiredoxin family protein/glutaredoxin	-1.69	nd	AHP1<br><br>GrxC	COG0678 [O]<br>COG0695

					[O]
NGO0934 YP_208042.1	hypothetical protein	-1.65	nd	SmtA	COG0500 [QR]
NGO1025 YP_208113.1	membrane protein	-1.56	nd	-	COG3198 [S]
NGO1055 YP_208138.1	acyl-CoA hydrolase	-1.78	nd	-	COG1607 [I]
NGO1130 YP_208209.1	phage associated protein	-1.61	nd	-	-
NGO1134 [*ribA*] YP_208213.1	GTP cyclohydrolase II	-1.75	nd	RibA	COG0807 [H]
NGO1196 YP_208268.1	ATP-dependent DNA helicase-like protein	-1.50	nd	HrpA	COG1643 [L]
NGO1308 YP_208372.1	guanosine-3',5'-bis(diphosphate) 3'-pyrophosphohydrolase	-1.56	nd	SpoT	COG0317 [TK]
NGO1427 YP_208482.1	transcriptional regulator	-1.74	nd	-	COG2932 [K]
NGO1428	hypothetical protein	-1.59	nd	-	-
NGO1442 YP_208496.2	alcohol dehydrogenase	-2.11	nd	AdhP	COG1064 [R]
NGO1451 YP_208505.1	hypothetical protein	-1.54	nd	-	-
NGO1455 YP_208508.1	membrane protein	-1.68	nd	MntH	COG1914 [P]
NGO1751 YP_208784.1	hypothetical protein/ putative NADH dehydrogenase I chain A	-1.54	nd	NuoA	COG0838 [C]
NGO1767 YP_208798.1	catalase protein KatA	-2.13	nd	KatE	COG0753 [P]
NGO1771 YP_208802.1	membrane protein	-3.19	nd	MscS	COG0668 [M]

NGO1778 [*aat*] YP_208809.1	leucyl/phenylalanyl-tRNA--protein transferase	-1.60	nd	Aat	COG2360 [O]
NGO1878 YP_208911.1	hypothetical protein	-2.08	nd	RimI	COG0456 [R]
NGO1931 YP_208956.1	GapC protein (glyceraldehyde 3-phosphate dehydrogenase C)	-1.59	nd	GapA	COG0057 [G]
NGO2182 [*rpmH*] YP_209187.1	50S ribosomal protein L34	-1.54	nd	RpmH	COG0230 [J]

Table S2B

**Gene [name] Protein ID**	**Description**	**Microarray mean fold change**	**qRT-PCR mean fold change**	**COG name**	**COG accession [category]**
**Upregulated**					
NGO0143 YP_207313.1	Na+/H+ antiporter	2.14	2.77	NhaC	COG1757 [C]
NGO0468 YP_207618.1	phage associated protein	2.32	nd	-	-
NGO0469 YP_207619.1	phage associated protein	2.44	nd	-	-
NGO0474 YP_207624.1	phage associated protein	2.42	nd	-	-
NGO0486 YP_207636.1	phage associated protein	2.33	nd	-	-
NGO0503 YP_207653.1	phage associated protein	2.25	nd	-	-
NGO0574 YP_207719.1	Cah-Carbonic anhydrase	9.63	15.8	Cah	COG3338 [P]
NGO0606	sodium-dependent	3.64	nd	COG0	COG0733

YP_207746.1	transport protein/ Transporter			733	[R]
NGO0626 YP_207766.1	murein transglycosylase	2.04	1.89	MltB	COG2951 [M]
NGO0758 YP_207885.1	ribulose-phosphate 3-epimerase (EC 5.1.3.1)	2.23	nd	Rpe	COG0036 [G]
NGO0762 YP_207889.1	hypothetical protein	2.47	nd	-	-
NGO0834 YP_207955.1	hypothetical protein/uncharacterized protein CsgG	2.07	4.72	CsgG	COG1462 [M]
NGO0835 YP_207956.1	genome-derived Neisseria antigen 1162/protein=GNA1162	2.20	3.15	COG4380	COG4380 [S]
NGO0897 YP_208008.1	hypothetical protein	2.02	nd	-	-
NGO1037 YP_208123.1	hypothetical protein	2.20	nd	-	-
NGO1064 YP_208146.1	putative carbon starvation protein	3.14	nd	CstA	COG1966 [T]
NGO1128 YP_208207.1	phage associated protein	2.40	nd	-	-
NGO1247 YP_208318.1	putative CDP-diacylglycerol transferase (EC 2.7.8.5)	3.07	nd	PgsA	COG0558 [I]
NGO1327 YP_208389.1	hypothetical protein	2.18	nd	-	-
NGO1393 YP_208453.1	MafA-like adhesin/MafA2	2.91	8.14	-	-
NGO1414 YP_208470.1	Na(+)-translocating NADH-quinone reductase subunit B (EC 1.6.5.-)	2.23	nd	NqrB	COG1805 [C]

NGO1461 YP_208517.1	hypothetical protein	2.37	nd	-	-
NGO1557 YP_208602.1	hypothetical protein	2.76	nd	-	-
NGO1585 YP_208626.1	MafB-like adhesin/ MafB4	2.80	4.9	-	-
NGO1681 YP_208718.1	hypothetical protein	3.76	nd	-	-
NGO1682 YP_208719.1	EmrB/QacA subfamily multidrug transporter	4.62	7.80	ProP	COG0477 [GEPR]
NGO1683 YP_208720.1	multi drug resistance resistance protein	2.46	8.16	EmrA	COG1566 [V]
NGO1751 YP_208784.1	NADH-quinone oxidoreductase subunit A (EC 1.6.99.5)	2.04	nd	NuoA	COG0838 [C]
NGO2009 YP_209029.1	hydroxymethylpyrimidine transporter CytX	3.27	nd	CodB	COG1457 [F]
NGO2011 YP_209031.1	ABC amino acid transporter/permease	3.02	nd	HisM	COG0765 [E]
NGO2012 YP_209032.1	ABC amino acid transporter/permease,	2.96	nd	HisM	COG0765 [E]
NGO2013 YP_209033.1	ABC transporter ATP-binding protein, amino acid	2.18	nd	GlnQ	COG1126 [E]
**Downregulated**					
NGO0114 YP_207286.1	glutaredoxin	-2.03	nd	GrxC	COG0695 [O]
NGO0116 YP_207288.1	preprotein translocase subunit SecB/Protein-export protein SecB	-2.12	nd	SecB	COG1952 [U]
NGO0322 YP_207481.1	hypothetical protein	-2.06	nd	Pyridox_ox_	COG3467 [R]

				2 super family	
NGO0420 YP_207575.1	hypothetical protein	-2.16	nd	COG3471	COG3471 [S]
NGO0614 YP_207753.1	ribonucleotide-diphosphate reductase subunit alpha	-2.07	nd	NrdA	COG0209 [F]
NGO1439 YP_208493.1	ABC transporter ATP-binding protein/ Macrolide export ATP-binding/permease protein MacB	-4.17	0.17	SalX	COG1136 [V]
NGO1440 YP_208494.1	ABC transporter periplasmic protein	-3.47	0.22	AcrA	COG0845 [M]
NGO1684 YP_208721.1	7-cyano-7-deazaguanine reductase/ /NADPH-dependent 7-cyano-7-deazaguanine reductase	-2.74	nd	COG0780	COG0780 [R]
NGO1779 YP_208810.1	Ferric uptake regulation protein	-2.21	0.61	Fur	COG0735 [P]
NGO1981 YP_209001.1	hypothetical protein	-3.22	nd	-	-
NGO1919 [*pgk*] YP_208944.1	phosphoglycerate kinase/Probable ATP-dependent permease	-2.24	nd	Pgk	COG0126 [G]

nd- not determined

a

b

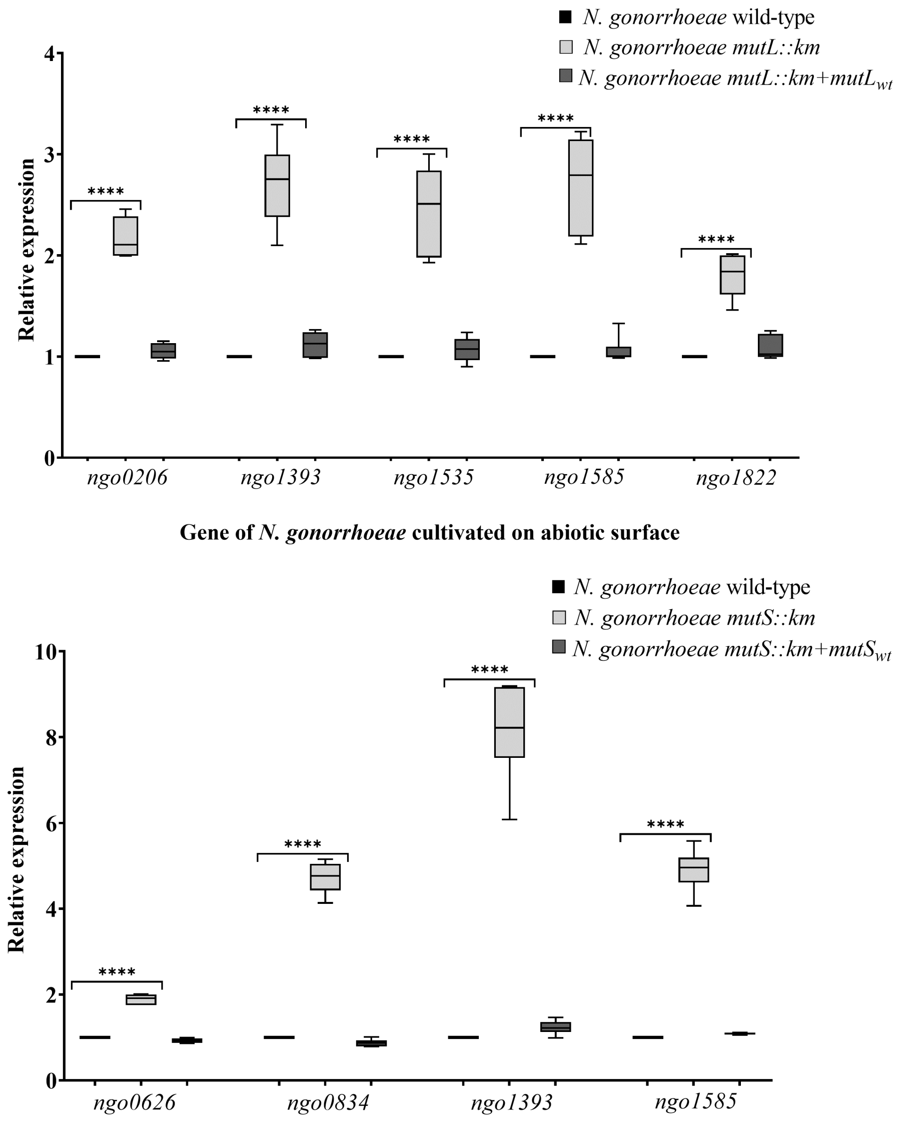

Figure S1. The expression of gonococcal genes encoding proteins that can influence adhesion and biofilm formation in (a) *N. gonorrhoeae mutL::km* and (b) *N. gonorrhoeae mutS::km* mutants cultivated on abiotic surface. The expression of the genes was evaluated by RT-qPCR and relative quantitation data analysis performed using the comparative quantification method ΔΔCt with 16S rRNA as the endogenous reference. The statistically significant differences between *N. gonorrhoeae mutL* and *mutS* mutants, the complementation strains (*N. gonorrhoeae mutL::km+mutL*$_{wt}$, *N. gonorrhoeae mutS::km+mutS*$_{wt}$) and wild-type were calculated using two-way ANOVA followed by Bonferroni post-tests. **** asterisks indicate *p* value <0.0001. Data represent mean values (± SD) from three independent experiments performed in triplicate.

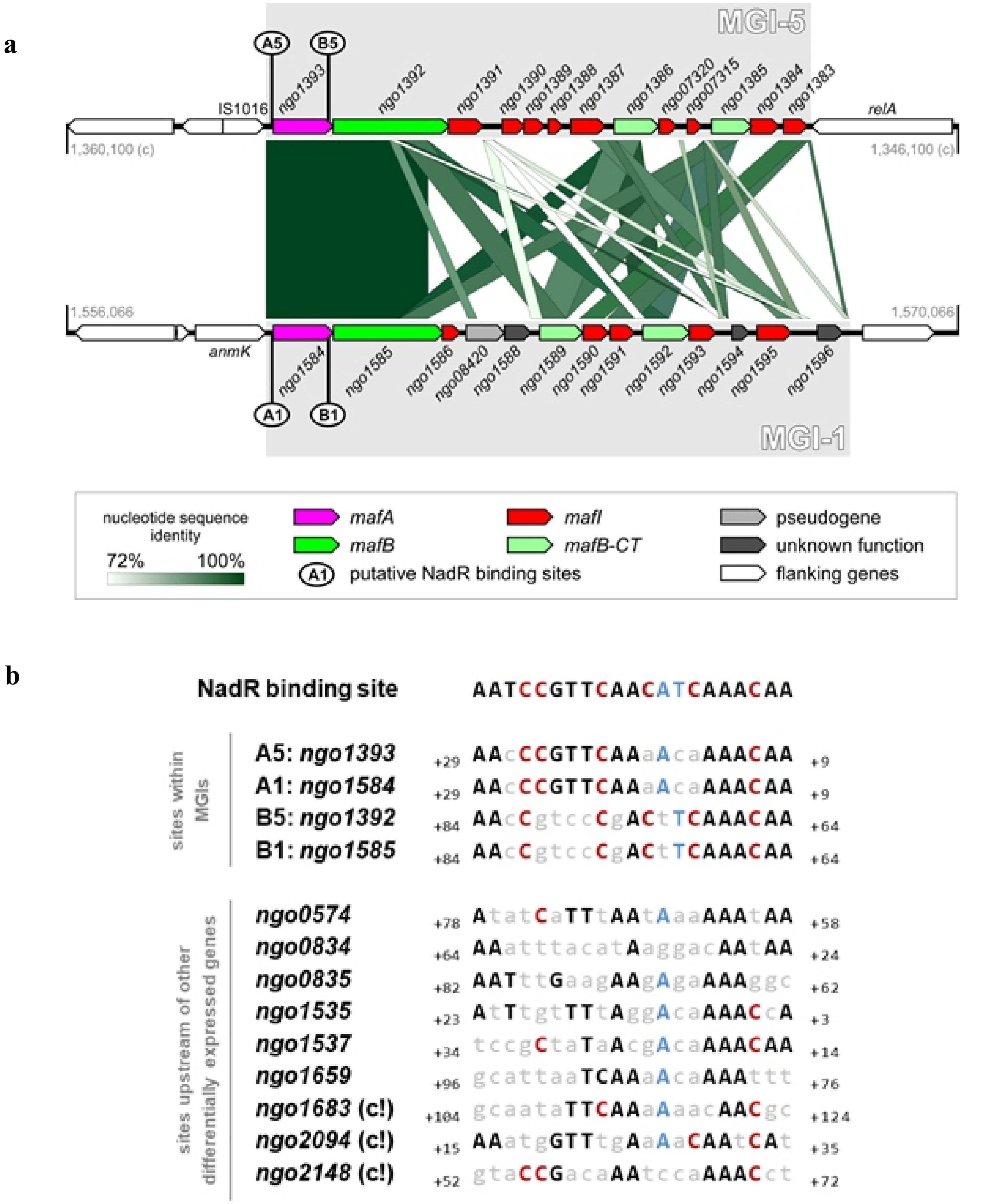

Figure S2. The organization of the two *N. gonorrhoeae* FA1090 *maf* genomic islands and putative NadR binding sites therein. (b) Nucleotide sequence comparison between MGI-5FA1090 and MGI-1FA1090. Genome comparisons were generated using BLASTn implemented in Easyfig 2.2.2 with 80% cutoff value. Key to the color code used to represent the genes within MGIs is given in the legend. (b) Comparison of the *N. meningitidis* MC58 mafA-associated NadR binding site with the sequences identified upstream of *mafA* and *mafB* genes in the *N. gonorrhoeae* FA1090 MGIs. For comparison, somewhat similar sequences found upstream of other genes differentially expressed in the analyzed *mutL::km* and *mutS::km* mutants are shown. Nucleotides essential for NadR binding in *N. meningitidis* MC58 are shown in black, those affecting affinity in blue, and those with no effect in binding activity in red. Numbers indicate the position relative to the first nucleotide of the start codon of each gene. Symbol “(c!)” indicates that the sequence is found in reverse orientation upstream of a given gene.

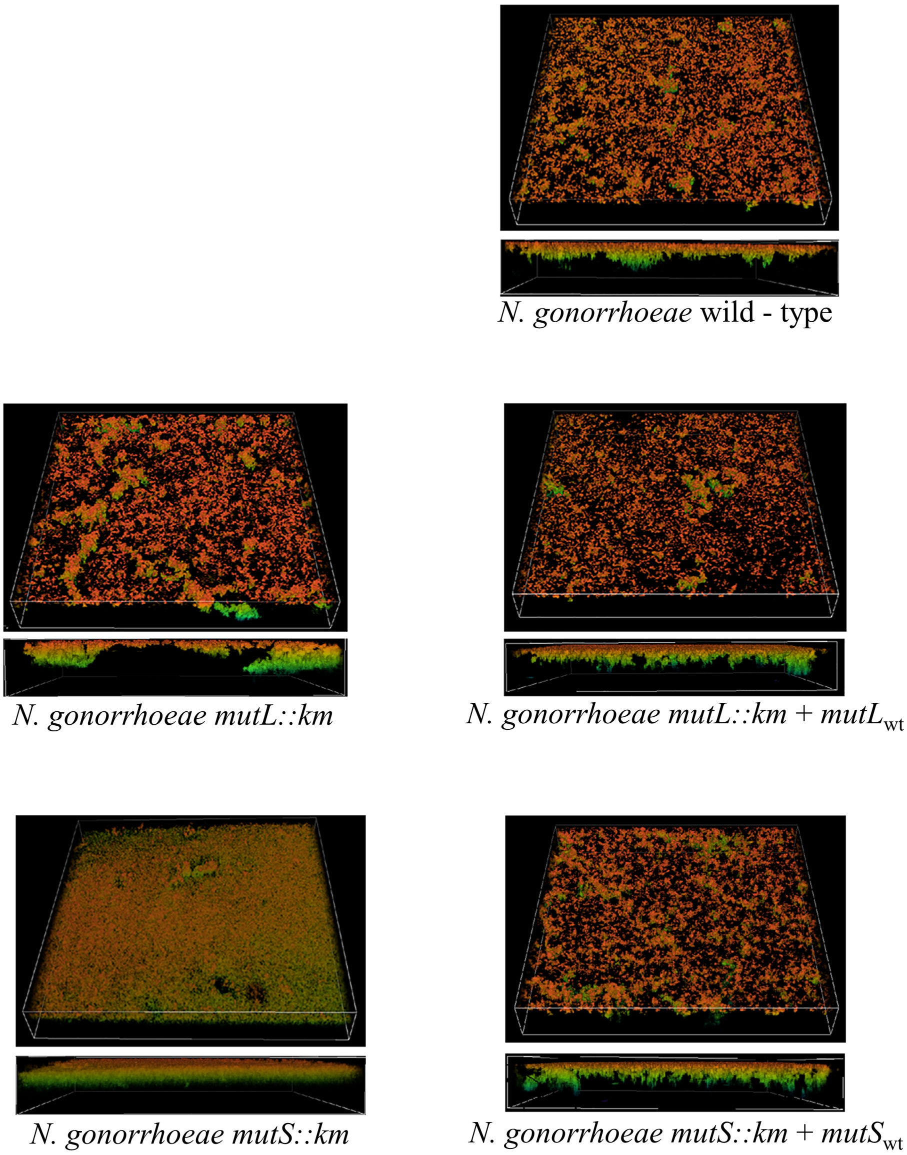

Figure S3. The structure and density of live biofilms formed by gonococci with a disrupted *mutL* or *mutS* gene (*N. gonorrhoeae mutL::km*, *N. gonorrhoeae mutS::km*, respectively), complementation strains (*N. gonorrhoeae mutL::km*+$mutL_{wt}$, *N. gonorrhoeae mutS::km*+$mutS_{wt}$) and the wild-type strain on abiotic surfaces assayed by SCLM. The live biofilms were stained with acridine orange and visualized by SCLM. The representative results from three independent experiments performed in triplicate are shown.

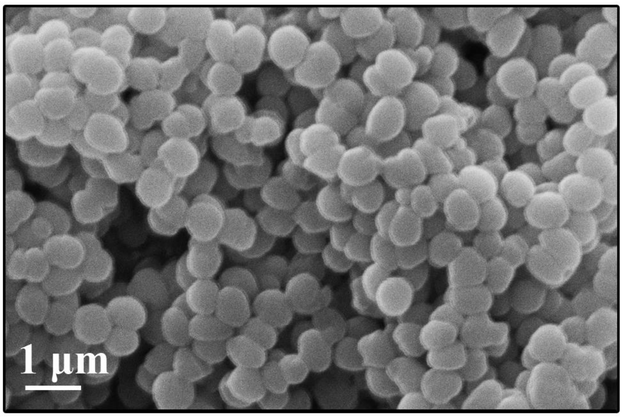

N. gonorrhoeae wild-type

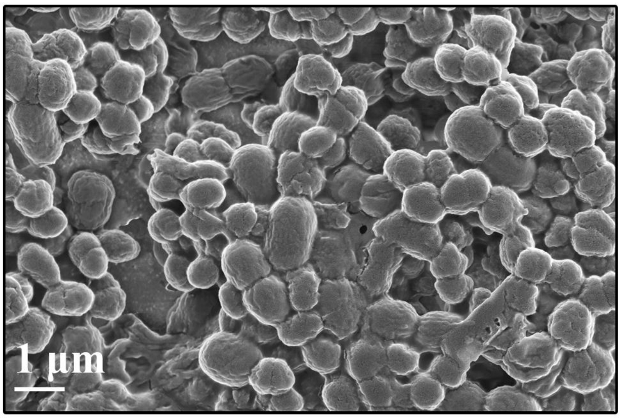

N. gonorrhoeae mutL::km

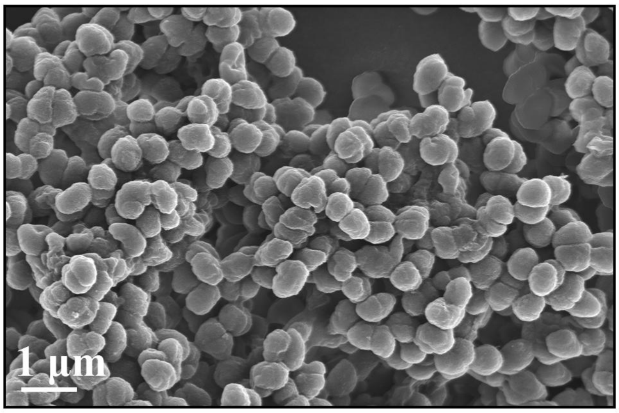

N. gonorrhoeae mutL::km + mutL$_{wt}$

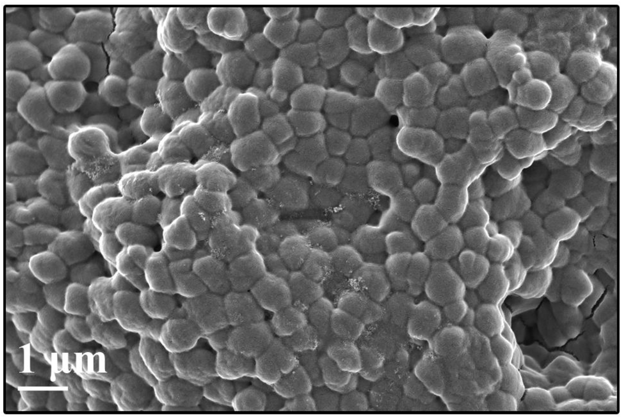

N. gonorrhoeae mutS::km

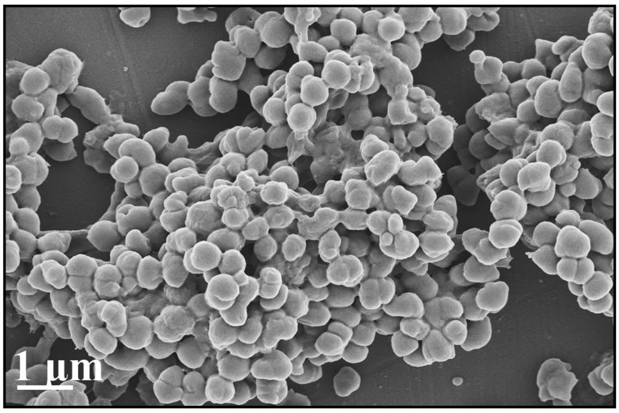

N. gonorrhoeae mutS::km + mutS$_{wt}$

Figure S4. The architecture of biofilms formed by gonococcal mutants with a disrupted *mutL* or *mutS* gene (*N. gonorrhoeae mutL::km*, *N. gonorrhoeae mutS::km*, respectively) in comparison with complementation strains (*N. gonorrhoeae mutL::km+mutL$_{wt}$*, *N. gonorrhoeae mutS::km+mutS$_{wt}$*) and the wild type on abiotic surface. The biofilms were cultivated on cover glasses at 37°C in 5% CO_2 for 24 h, fixed with glutaraldehyde and sodium cacodylate mixture, coated with gold-palladium and imaged by FE SEM (magnitude 25 000×). All experiments were performed in triplicate, and representative images are shown.

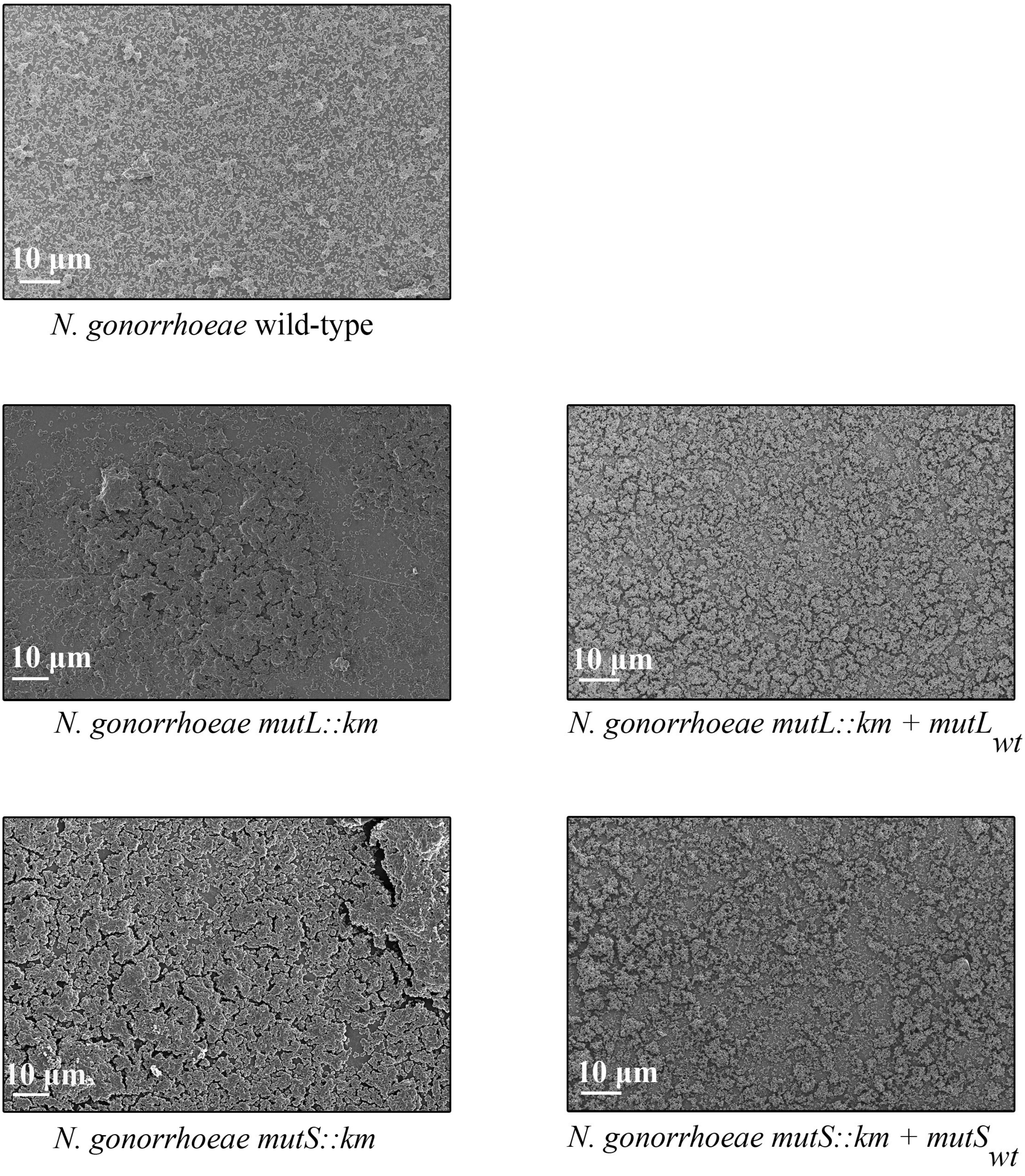

Figure S5. Visualization of biofilms formed by gonococcal mutants with a disrupted *mutL* or *mutS* gene (*N. gonorrhoeae mutL::km*, *N. gonorrhoeae mutS::km*, respectively) in comparison with complementation strains (*N. gonorrhoeae mutL::km+mutL$_{wt}$*, *N. gonorrhoeae mutS::km+mutS$_{wt}$*) and the wild type on abiotic surface. The biofilms were cultivated on cover glasses at 37°C in 5% CO_2 for 24 h, fixed with glutaraldehyde and sodium cacodylate mixture, coated with gold-palladium and imaged by FE SEM (magnitude 2500 ×). All experiments were performed in triplicate, and representative images are shown.

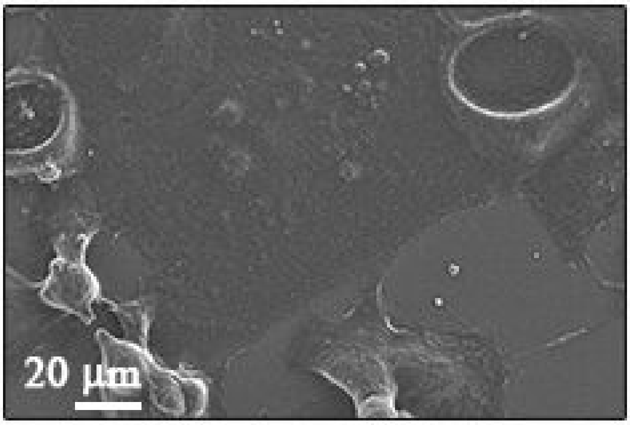

uninfected epithelial cells

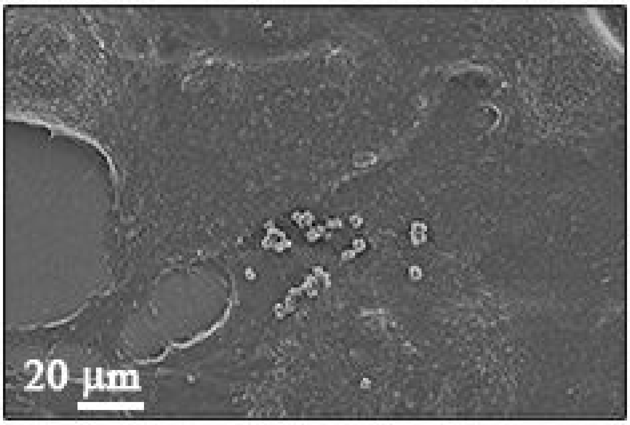

N. gonorrhoeae wild-type

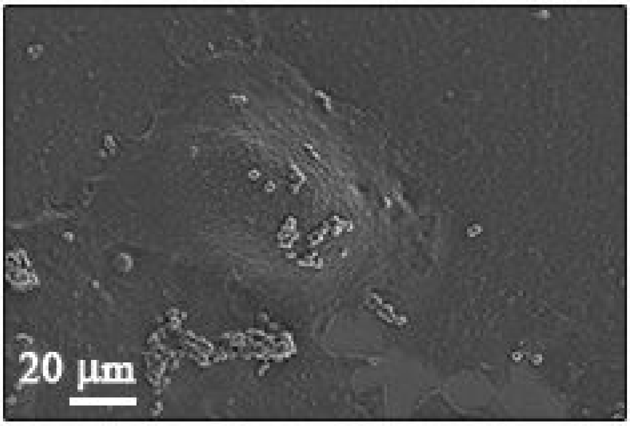

N. gonorrhoeae mutL::km

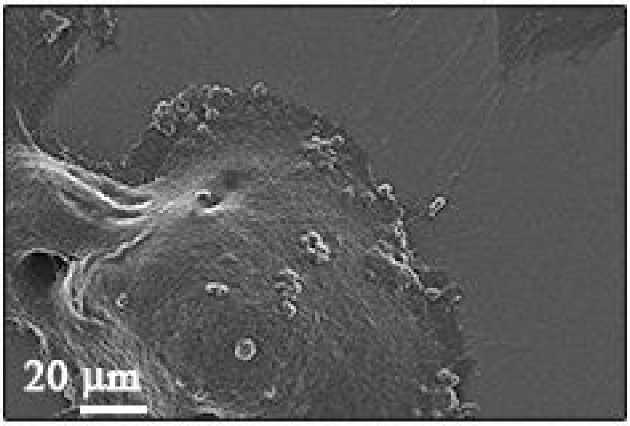

N. gonorrhoeae mutL::km + $mutL_{wt}$

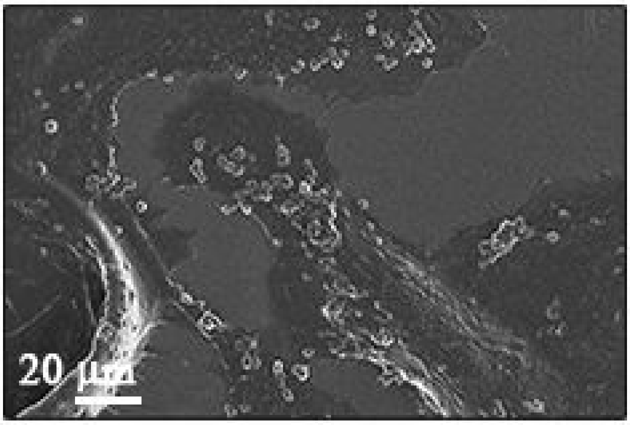

N. gonorrhoeae mutS::km

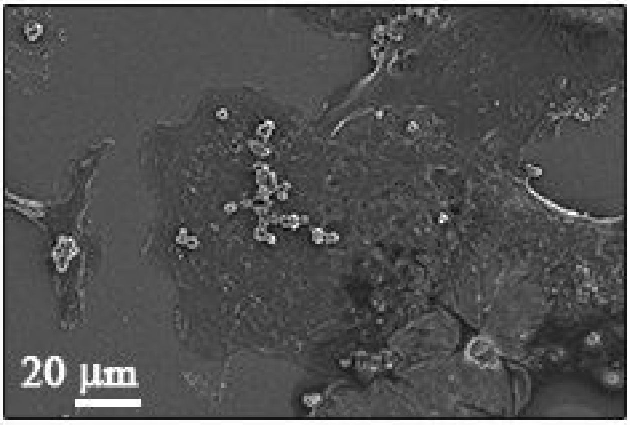

N. gonorrhoeae mutS::km + $mutS_{wt}$

Figure S6. Microscopy visualization of *N. gonorrhoeae mutL::km* and *N. gonorrhoeae mutS::km* attached to epithelial cells in comparison to the complementation (*N. gonorrhoeae mutL::km*+$mutL_{wt}$ and *N. gonorrhoeae mutS::km*+$mutS_{wt}$) and wild-type strains assayed by FE SEM. *N. gonorrhoeae* was added to the epithelial cells and cultivated for 4 h, followed cells were fixed with a glutaraldehyde and sodium cacodylate mixture, coated with gold-palladium and visualized by FE SEM (magnitude 2500×). All experiments were performed in triplicate, and representative images are shown.
